# Improved Strain Transfer Efficiency in Large-Area Two-Dimensional MoS_2_ Obtained by Gold-Assisted Exfoliation

**DOI:** 10.1021/acs.jpclett.4c00855

**Published:** 2024-06-10

**Authors:** Álvaro Rodríguez, Onur Çakıroğlu, Hao Li, Felix Carrascoso, Federico Mompean, Mar Garcia-Hernandez, Carmen Munuera, Andres Castellanos-Gomez

**Affiliations:** Materials Science Factory, Instituto de Ciencia de Materiales de Madrid (ICMM)−Consejo Superior de Investigaciones Científicas (CSIC), C. Sor Juana Inés de la Cruz, 3, 28049 Madrid, Spain

## Abstract

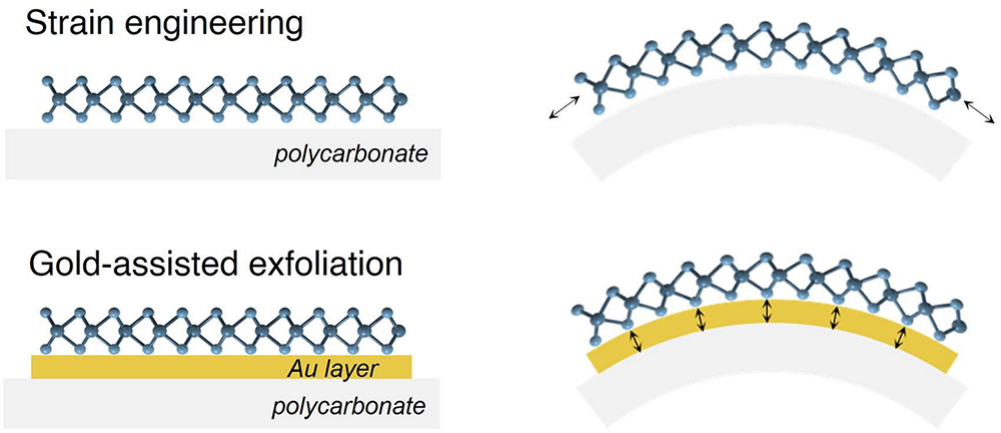

Strain engineering represents a pivotal approach to tailoring the optoelectronic properties of two-dimensional (2D) materials. However, typical bending experiments often encounter challenges, such as layer slippage and inefficient transfer of strain from the substrate to the 2D material, hindering the realization of their full potential. In our study, using molybdenum disulfide (MoS_2_) as a model 2D material, we have demonstrated that layers obtained through gold-assisted exfoliation on flexible polycarbonate substrates can achieve high-efficient strain transfer while also mitigating slippage effects, owing to the strong interfacial interaction established between MoS_2_ and gold. We employ differential reflectance and Raman spectroscopy for monitoring strain changes. We successfully applied uniaxial strains of up to 3% to trilayer MoS_2_, resulting in a notable energy shift of 168 meV. These values are comparable only to those obtained in encapsulated samples with organic polymers.

Tuning the optoelectronic properties of two-dimensional (2D) materials via strain engineering is a widely adopted strategy to tailor the properties of these materials.^1−3^ Many approaches have been proposed, such as applying tensile strain through substrate bending,^4−7^ inducing biaxial strain via the thermal expansion of a substrate,^8,9^ or generating strain externally through contaminants, like nanobubbles, wrinkles, and pillars, among other innovative ideas.^10−15^ In general, using a flexible substrate is the most common approach to transferring strain to the 2D material. Uniaxial tensile or compressive strain can be applied to the substrate using bending setups, and *in situ* optical characterization can be performed through reflectance, photoluminescence (PL), or Raman spectroscopy.^5,16^ While the use of polymers with a high Young’s modulus can transfer strain more effectively, the interaction between the pristine polymer substrate and the van der Waals material is typically very weak, leading to issues, such as slippage and uneven distribution of the applied strain throughout the layer. New strategies to mitigate these conditions have been proposed, such as the use of a spin-coated poly(vinyl alcohol) (PVA) layer to enhance adhesion to the substrate^17^ or encapsulation with adamantane using remote plasma-assisted vacuum deposition to reduce slippage and enhance transfer efficiency.^18^ Both methods have been evidenced to successfully increase the maximum strain applied.

Gold-assisted exfoliation has proven to be a viable method for obtaining large-area monolayers (1L) of MoS_2_ as a result of the strong interaction between the first MoS_2_ layer and Au.^19−22^ Indeed, Au acts as a strong adhesive layer because of the formation of a moiré pattern between lattices of MoS_2_ and Au(111).^12,23^ The exfoliation yield is influenced by the duration of exposure of gold to the ambient environment and the surface roughness.^21,24^ Traditionally, SiO_2_/Si substrates have been the primary choice for the application of this method. However, the adoption of flexible substrates is essential for implementing strain. Although gold-assisted exfoliation has been demonstrated to successfully work with various substrates, including polymer substrates,^22^ the utilization of flexible substrates remains unexplored in practical applications, despite their potential for enabling strain engineering.

In this work, we utilize gold-assisted exfoliation of MoS_2_ on 250 μm thick polycarbonate (PC) substrates and observe an efficient transfer of strain through the use of a gold layer between PC and MoS_2_. The lateral dimensions of the flakes are in the order of several hundred micrometers and are determined by the size and quality of the original bulk crystal.^21,22^ We employ differential reflectance spectroscopy and Raman spectroscopy to monitor the strain.^25,26^ The application of uniaxial strain is facilitated by a motorized straining setup, enhancing the reproducibility of experiments.^27^ This setup enables the application of high levels of strain, up to 3% for 3L MoS_2_, and achieves energy shifts similar to those observed when using encapsulated 2D materials. Among the advantages of our methodology, our non-encapsulated approach would enable imaging with a scanning probe microscope to observe changes when applying high strain values. Additionally, in future strain sensors, an exposed surface facilitates the interaction with molecules or analytes and enables accessibility for surface reactions or modifications. Lastly, the possibility of transferring other exfoliated materials onto the MoS_2_–Au system holds promise for improving the substrate interaction through van der Waals forces.

*Identification of the Number of Layers of MoS_2_–Au on PC Substrates*. The strong interaction between transition metal dichalcogenides (TMDCs) and Au influences the optical properties of the resulting material. For example, the PL of monolayer (1L) MoS_2_ directly exfoliated on Au is significantly quenched compared to free-standing MoS_2_.^28,29^ Additionally, its Raman spectrum exhibits characteristic broadening and shifting of the E and A_1_ Raman modes as a result of strain and doping effects.^24,29^ We use E and A_1_ notation for in-plane and out-of-plane Raman modes instead of the commonly used E′ and A′_1_ notation for freestanding 1L MoS_2_ (or E^1^_2g_ and A_1g_ for bulk MoS_2_) as a result of the symmetry reduction occurring in the 1L MoS_2_–Au heterostructure from D_3h_ to C_3v_.^28^

Figure 1a shows the optical image of a MoS_2_ sample directly exfoliated on Au-covered PC. For the sake of comparison, we use thicknesses of 3 nm for Ti and 6 nm for Au in all measurements. We usually obtain a continuous 1L MoS_2_ of several tens of micrometers, confirming the flatness of the PC substrate. We conducted a texture analysis of the preferred orientations of Au grains along the normal direction of the sample using X-ray diffraction and found that the most abundant crystallographic orientation for the evaporated polycrystalline Au films is along the[111] direction (see the inset of Figure 1a). This method facilitates the preferential exfoliation of large 1L MoS_2_ areas on the Au surface while also producing thicker layers, such as bilayers (2L) and trilayers (3L). Apart from the assignment with optical microscopy, we employ Raman spectroscopy to unequivocally determine the number of layers. As mentioned before, both E and A_1_ modes experience peak shifts in 1L MoS_2_–Au. On one hand, the E mode broadens and red shifts, attributed to the tensile strain resulting from the lattice mismatch between 1L MoS_2_ and Au(111).^24,29^ On the other hand, a low-frequency A_1_ mode (at 396.4 cm^–1^) emerges as a result of the strong interaction with Au, which is usually seen as a splitting of the A_1_ mode. These two effects are less visible in 2L MoS_2_ and practically vanished in 3L MoS_2_. All of the Raman features are reproducible in our samples on PC, as seen in Figure 1b.

**Figure 1:**
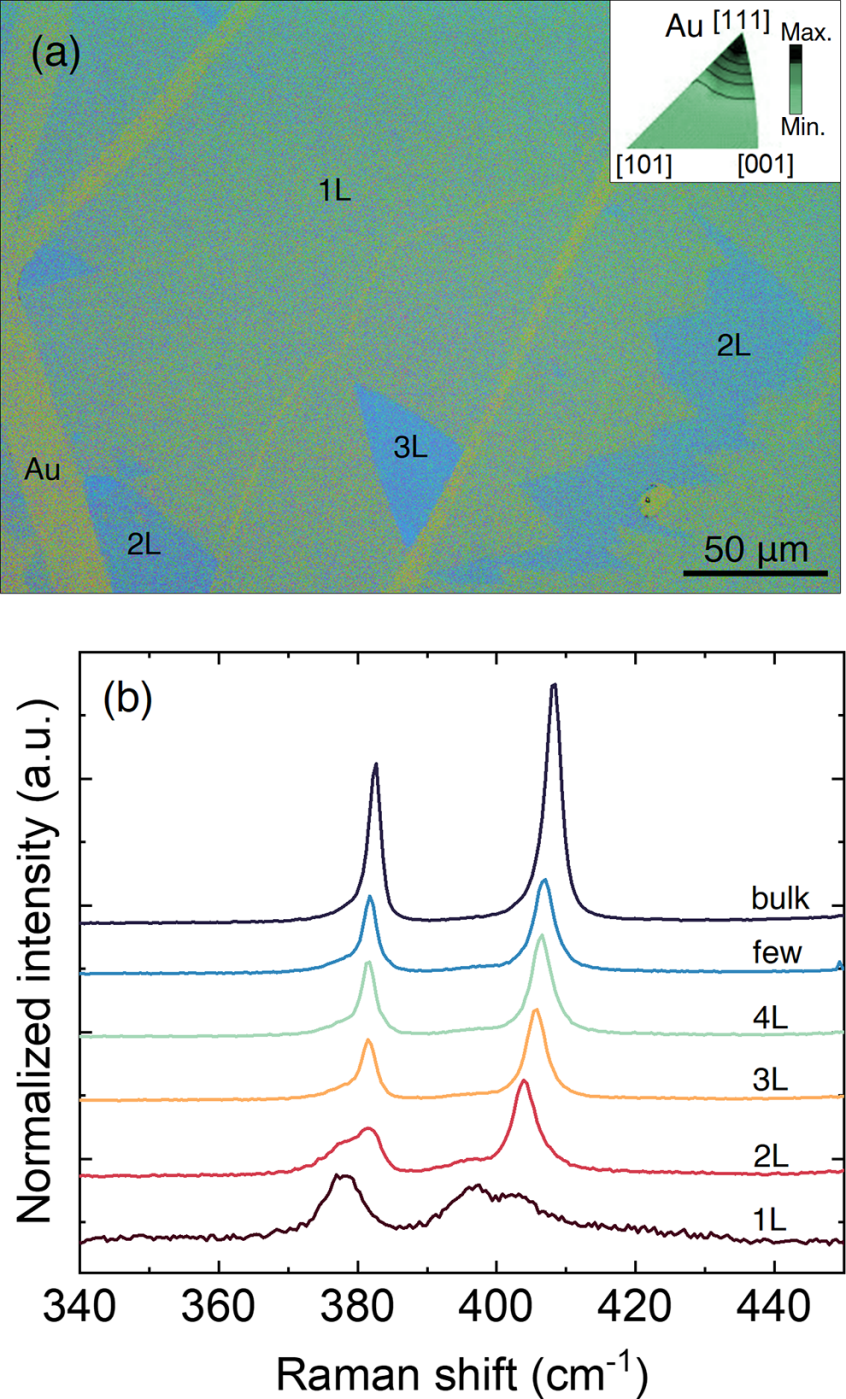
(a) Optical image of MoS_2_ exfoliated on Au-covered PC substrates. The inset shows the preferential orientation of Au crystals in the e-beam-evaporated films obtained by X-ray diffraction. (b) Raman spectra of MoS_2_–Au as a function of the number of layers. Additional optical images of the samples are shown in Figure S1 of the Supporting Information.

*Layer Identification Using Differential Reflectance Spectroscopy*. To further advance our analysis, we carried out differential reflectance spectroscopy measurements, confirming that this technique can also be employed to determine the number of layers in MoS_2_–Au. Figure 2a presents representative spectra for different numbers of layers. Two distinct bands, located at approximately 1.88 and 2.02 eV, corresponding to the A and B excitons, respectively, are visible and become sharper with increasing thickness.^30,31^Figure 2b displays the average energies of the A and B excitons for several samples. The exciton energy for 1L is shifted in comparison to MoS_2_ layers transferred onto PC without the presence of Au. Specifically, for 1L MoS_2_, the A and B excitons, centered at 1.905 and 2.03 eV^26^ on bare polydimethylsiloxane (PDMS) substrates, experience a red shift to 1.86 and 1.98 eV on Au-covered substrates. This shift is consistent with the appearance of tensile strain in the 1L MoS_2_–Au heterostructure, as previously determined by Raman spectroscopy. It has been previously shown that the E mode can be shifted by up to 7 cm^–1^ compared to 1L MoS_2_ on Si–SiO_2_ substrates.^24,29^ As the number of layers increases, the contribution of the substrate becomes less significant, and the exciton energy for a larger number of layers resembles the energy observed when using substrates with the absence of Au.^26^

**Figure 2:**
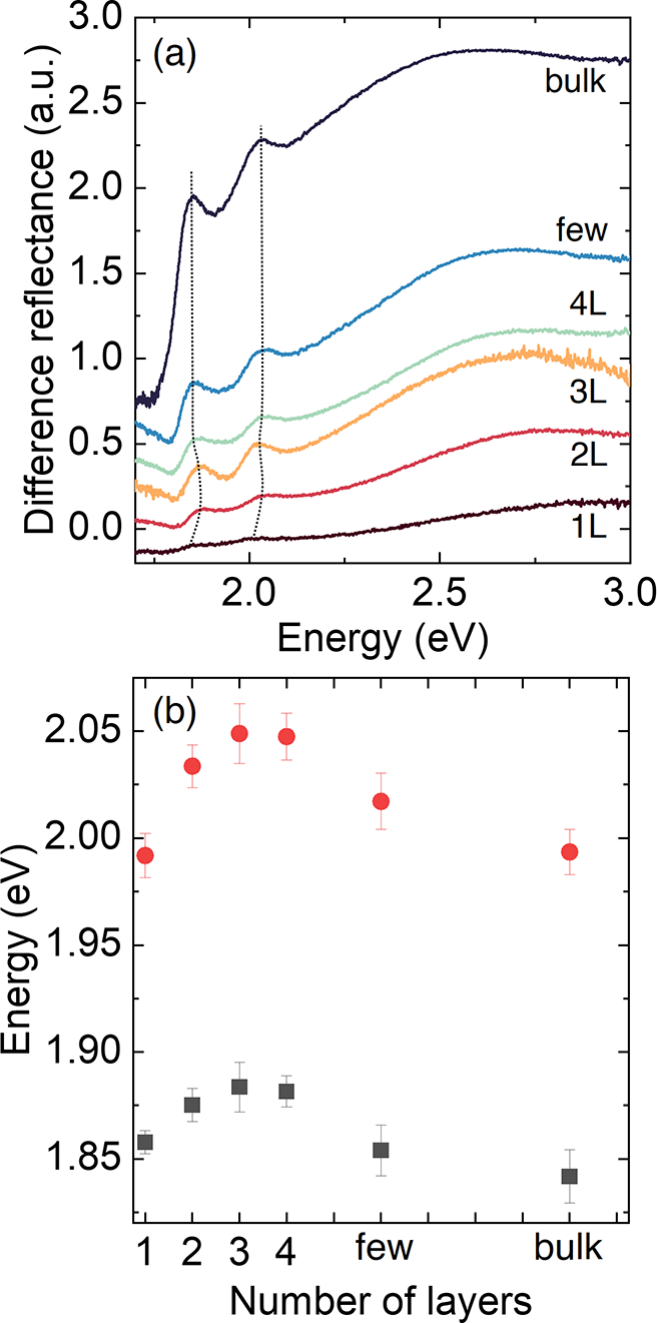
Differential reflectance spectroscopy of MoS_2_ directly exfoliated on Au-covered PC. (a) Differential reflectance spectra of MoS_2_–Au with an increasing number of layers. Dash lines indicate the maximum of the A exciton peak for different number layers. (b) Variation of A (black squares) and B (red circles) exciton energies as a function of the number of layers. Error bars were obtained from three different samples of each thickness.

*Uniaxial Strain of MoS_2_–Au on PC Substrates: Strain-Dependent Differential Reflectance Spectroscopy*. As a consequence of the strong interfacial interaction between MoS_2_ and Au, an efficient transfer of strain from the substrate to MoS_2_ is expected. We employ an automated three-point bending setup to apply a uniaxial strain. This setup allows us to apply strain with high accuracy and precision. Detailed information about the setup can be found in a previous work.^27^ Differential reflectance spectra as a function of the strain for 1L, 2L, and 3L MoS_2_ are shown in panels a–c of Figure 3. Both A and B excitons red shift when increasing the strain and stop shifting at high strain values, corresponding to the point of slippage of the layers. Thicker MoS_2_ layers, for instance, 3L MoS_2_, can withstand up to 3% strain. Additional samples can be found in Figures S2–S4 of the Supporting Information, including an example showing the point of slippage of 3L MoS_2_ beyond 3%.

**Figure 3:**
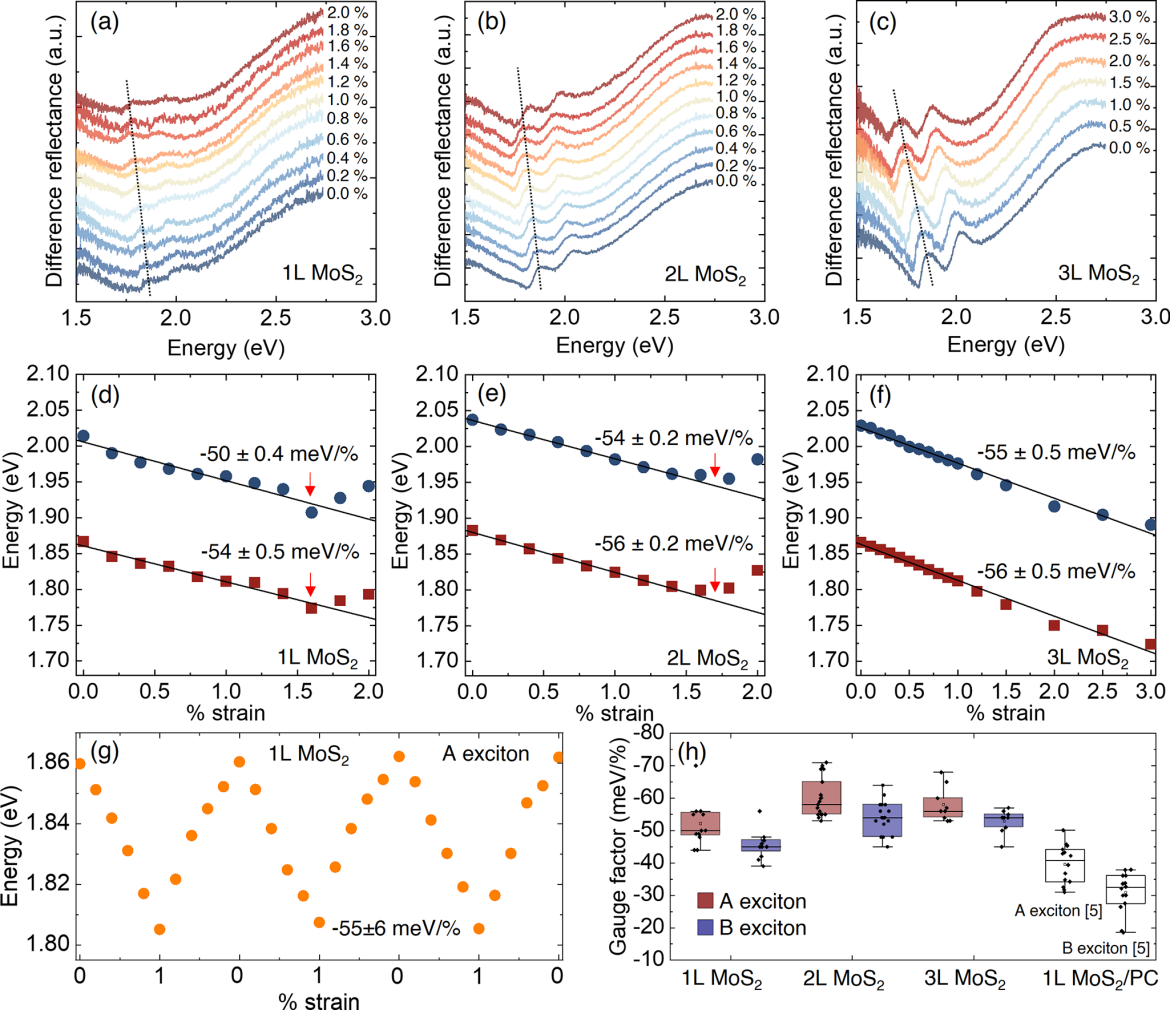
Observation of changes in MoS_2_–Au with differential reflectance under uniaxial strain. Differential reflectance spectra of (a) 1L MoS_2_–Au, (b) 2L MoS_2_–Au, and (c) 3L MoS_2_–Au under different applied strains. Different colors represent the different strain values, as indicated in the figure. Dash lines indicate the maximum of A exciton peak for increasing strain values. The spectra are shifted vertically for better visibility. Gauge factors obtained for A (dark red squares) and B (dark blue circles) exciton energies as a function of the applied uniaxial strain: (d) 1L MoS_2_–Au, (e) 2L MoS_2_–Au, and (f) 3L MoS_2_–Au. Black solid lines are the linear regression of the A and B exciton energies at different strain values. Red arrows indicate the point of slippage. (g) A exciton energy for different strain cycles between 0 and 1%. (h) Statistics of the gauge factors obtained on different MoS_2_–Au samples (12 samples of 1L, 16 samples of 2L, and 10 samples of 3L). Statistics of 1L MoS_2_ on PC without Au are shown for comparison.

We calculate the differential reflectance gauge factor (the shift of the A and B exciton energies in the differential reflectance spectra per percentage of uniaxial strain) for 1L, 2L, and 3L MoS_2_ on Au and obtain gauge factors of −54, −56, and −56 meV/% for the A exciton and −50, −54, and −55 meV/% for the B exciton, respectively, as seen in panels d–f of Figure 3. The fitting curves can be found in Figure S5 of the Supporting Information. These values are higher than those obtained in pristine PC, which is −37 meV/% for A exciton of 1L MoS_2_ on PC using the same setup,^27^ indicating a more efficient strain transfer when the Au layer is used as an adhesive layer between PC and MoS_2_. The slippage of MoS_2_ flakes on the substrate is significantly reduced as a result of the strong interaction between MoS_2_ and Au. This interaction yields a maximum strain of 1.5% for 1L and about 2% for 2L and reaches a maximum of 3% strain in 3L MoS_2_. In contrast, for MoS_2_ flakes without the presence of Au, the maximum strain rarely surpasses 1.3%.^5^ This trend underscores the pronounced impact of the layer thickness on the MoS_2_–Au interaction. One possible explanation for this result could be the tensile pre-strain experienced by 1L MoS_2_ during the exfoliation process, as evidenced by Raman spectroscopy measurements.^24^

Figure 3g shows the variation of the A exciton upon applying different strain cycles for 1L MoS_2_. Strain values of up to 1% can be applied over multiple cycles without altering the initial position of the A exciton. We perform multiple relaxation cycles on 3L MoS_2_, applying strains of up to 3% to detect changes in peak positions. The spectra for each strain value and the positions of the A and B excitons are shown in Figure S7 of the Supporting Information. Finally, we measure a large number of samples (12 samples of 1L MoS_2_, 16 samples of 2L MoS_2_, and 10 samples of 3L MoS_2_) and summarize the gauge factors obtained for the different number of layers in Figure 3h, being −52 ± 7, −60 ± 6, and −58 ± 5 meV/% for A exciton and −46 ± 4, −54 ± 5, and −53 ± 4 meV/% for B exciton in 1L, 2L, and 3L MoS_2_, respectively. The values obtained without Au using the sample PC substrates are also shown for comparison.^5^ The improvement of the gauge factors obtained in the Au-exfoliated samples is notable.

*Raman Spectroscopy*. Raman spectroscopy is another powerful technique that can be used to determine the strain in 2D materials. As previously mentioned, the Raman spectrum of 1L MoS_2_ is characteristic of having the E mode already downshifted and a splitting of the A_1_ mode into two components, A_1_(L) and A_1_(H).^24,29^ L and H denote low and high frequency, respectively. When applying uniaxial strain, a break of the degeneration is expected and two E components should be observed.^32^Figure 4a shows the Raman spectra of 1L MoS_2_ for increasing strain values up to 1.5%. The deconvolution of the peaks is shown in Figure S6 of the Supporting Information. The positions of the peaks as a function of the strain are shown in Figure 4d. A small shift of the A_1_(H) mode is observed, and a slightly larger shift is observed for the A_1_(L) mode. This mode was assigned to the areas where MoS_2_ is in intimate contact with Au at the nanoscale level and is the fingerprint of the strong interaction of MoS_2_ and Au.^24,28^ Concerning the E mode, similar to the samples without the Au layer, the mode is split in two components at strain levels higher than 1%. The splitting is more evident in 2L (panels b and e of Figure 4) and 3L (panels c and f of Figure 4) MoS_2_. The Raman shift is linear up to 3% of applied strain for 3L MoS_2_. The large shifts observed in the Raman spectra support the reflectance measurements and confirm that a higher tunability of the bandgap is achieved in the samples with Au.

**Figure 4:**
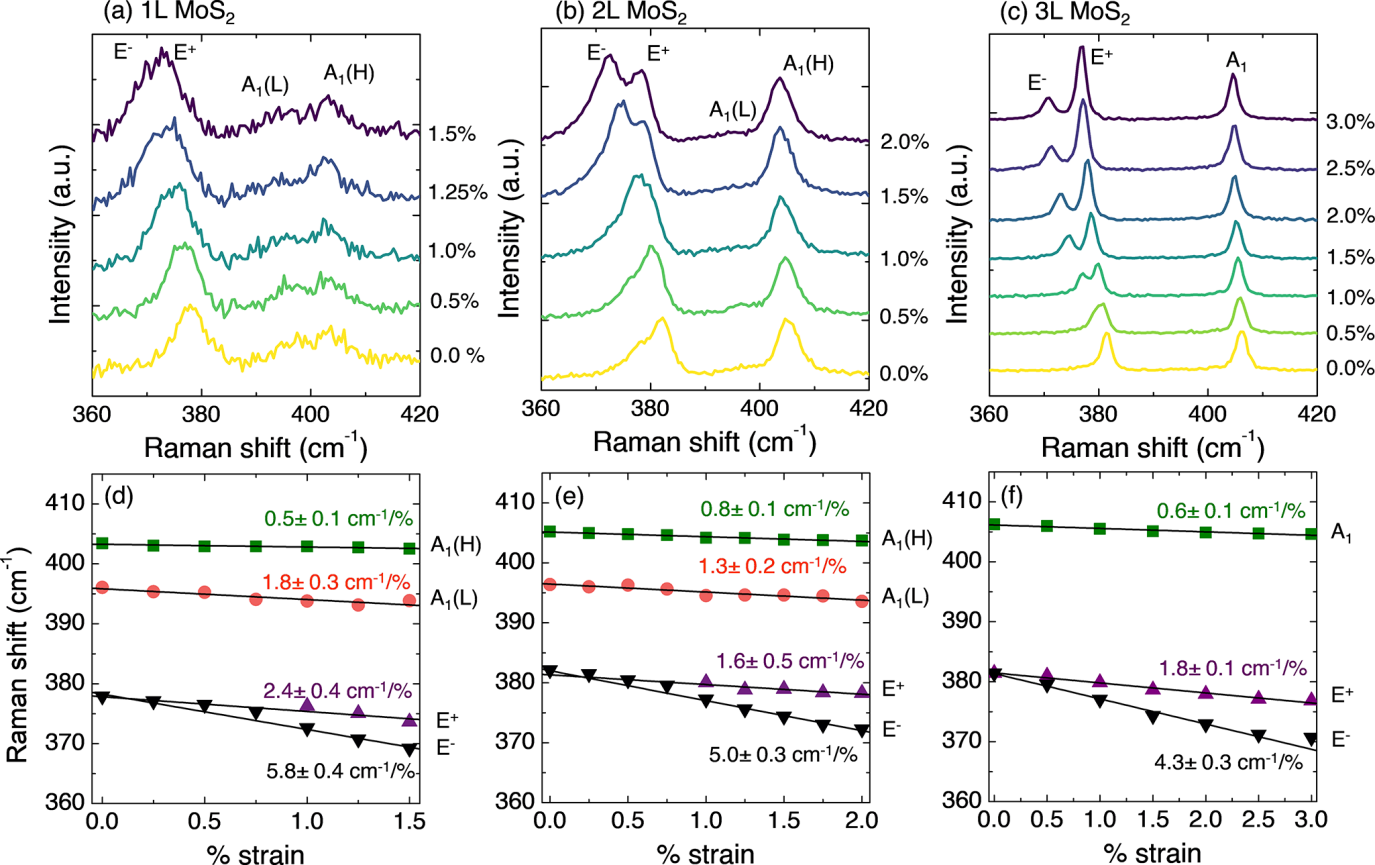
Observation of changes in MoS_2_–Au with Raman spectroscopy under uniaxial strain. Raman spectra of (a) 1L MoS_2_ and (b) 2L MoS_2_, and (c) 3L MoS_2_ under different applied strains. Different colors represent different strain values, as indicated in the figure. The assignment of the modes is indicated above the corresponding Raman peak. The spectra are vertically shifted for better visibility. Peak positions were extracted from the Raman peak fitting of (d) 1L MoS_2_, (e) 2L MoS_2_, and (f) 3L MoS_2_. The frequency shift of the Raman modes in the spectra per percentage of uniaxial tensile strain is indicated for each mode [A_1_(H), A_1_(L), E^+^, and E^–^].

*Comparison to Previous Literature*. Figure 5a presents a summary of gauge factors and maximum strains obtained through reflectance/absorbance or PL in various studies found in the literature.^33−38^ The reported values vary across different works as a result of differences in straining setups and substrates, which makes direct comparisons challenging. Noteworthy, references by Carrascoso et al.^5^ and Çakıroğlu et al.,^27^ indicated with red circles in Figure 5a, employed setups and polymer substrates very similar to those utilized in the present work.

**Figure 5:**
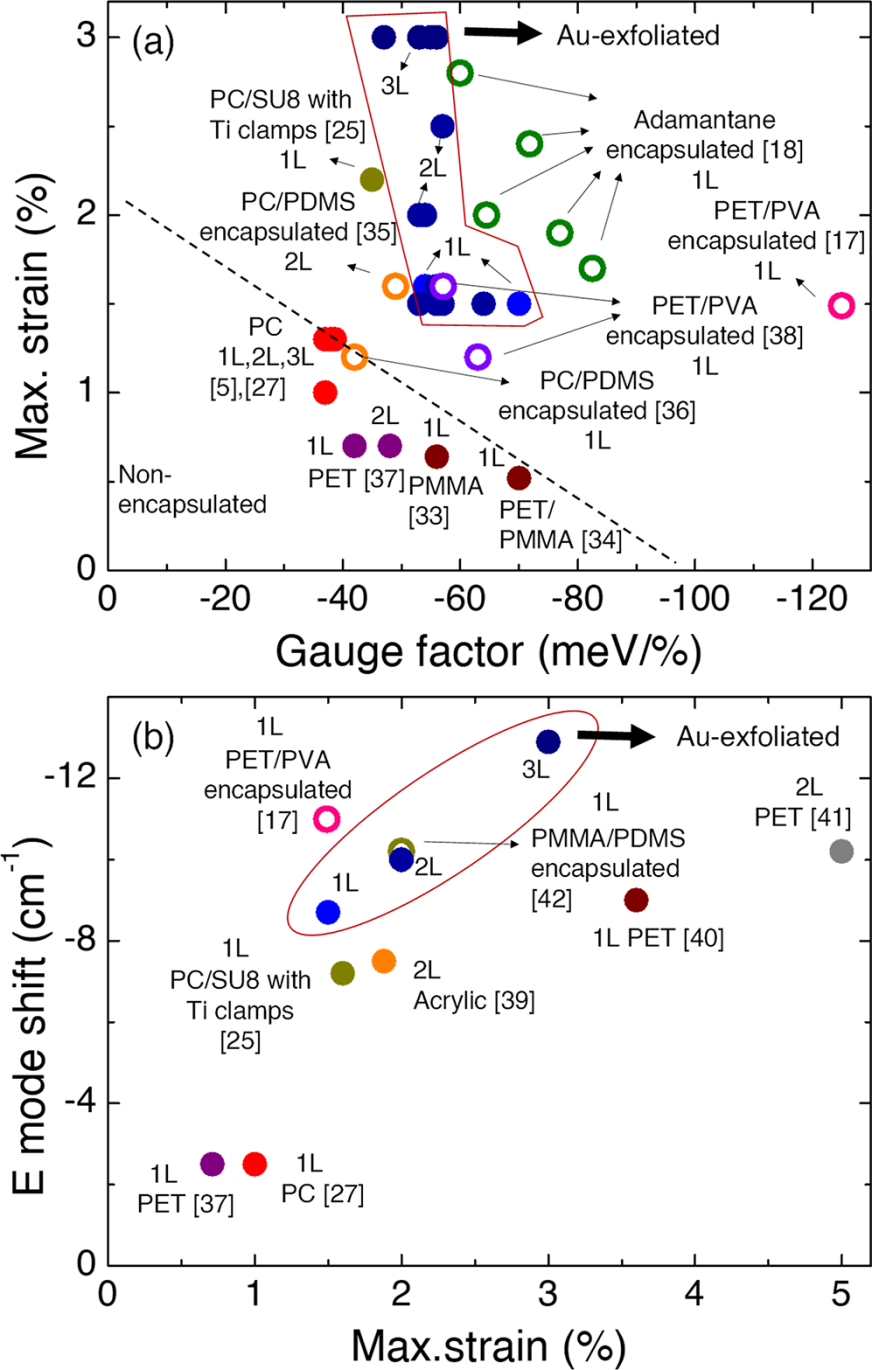
(a) Summary of the maximum strain and gauge factors reported on the A exciton of MoS_2_ upon applying uniaxial strain with reflectance and photoluminescence. (b) E mode shift observed by Raman spectroscopy versus maximum strain applied. Blue circular dots represent the data measured in this work, and the experimental data can be found in the Supporting Information. Results from encapsulated samples are represented by open circles.

In these mentioned works, gauge factors and maximum energy shifts (gauge factor × maximum strain) for the A exciton of −37, −38, and −38 meV/% and −48.5, −49.5, and −49.5 meV were obtained for 1L MoS_2_, 2L MoS_2_, and 3L MoS_2_, respectively. Comparing these findings to the results obtained in gold-assisted exfoliated layers (blue circles in Figure 5a), where gauge factors and energy shifts for the A exciton are −54, −56, and −56 meV/% and −86.4, −100.8, and −168 meV for 1L, 2L, and 3L MoS_2_, respectively, reveals a substantial improvement. The maximum shift nearly doubles in value for 1L MoS_2_ and shows even better results for 2L and 3L MoS_2_. Furthermore, it is noteworthy that both strain transfer efficiency and the maximum applied strain are higher when utilizing Au-covered substrates.

The studies by Li et al.^17^ and Carrascoso et al.^18^ have reported gauge factor values surpassing those obtained in this work, as indicated in the graph with open circles. In those works, the researchers employed encapsulation of MoS_2_ layers to enhance the strain applied. It is worth noting that, while PVA encapsulation holds the potential to achieve gauge factors exceeding 120 meV/% in monolayer MoS_2_,^17^ previous attempts to replicate similar results have been unsuccessful.^18^ Interestingly, the same authors utilized the same protocol but only managed to obtain gauge factors in the range of 60 meV/% for monolayer MoS_2_.^38^ This discrepancy underscores the challenges in achieving consistent results with spin-coated encapsulation techniques and suggests the need for further investigation into optimizing the process. In contrast, our study achieves comparable values without encapsulation and on significantly larger sample areas, attributed to the robust interaction between MoS_2_ and Au. Reaching significant high strain values while maintaining a free surface would enable imaging using a scanning probe microscope, facilitating the measurement of topographical changes and various properties at the nanoscale under high-strain conditions.

The summary of the values obtained with Raman spectroscopy is depicted in Figure 5b.^39−42^ The observed shift for the E mode is among the largest measured with Raman spectroscopy, particularly notable in the case of 3L MoS_2_. This finding confirms the enhanced efficiency of strain transfer in MoS_2_ exfoliated on Au. The improvement compared to that of bare PC (red circles) is remarkable, with only encapsulation achieving similar values. The implications of our research extend beyond MoS_2_, as the proposed methodology can be applied to various 2D materials, opening avenues for the development of strain-engineered flexible devices with enhanced performance and functionality.

We have shown that the gold-assisted exfoliation of MoS_2_ on flexible substrates enables the fabrication of large MoS_2_ layers with effective strain transfer capabilities. In fact, using a gold interfacial layer improves the efficiency of the strain transfer as a result of the strong interaction and bonding between Au and MoS_2_. Reflectance spectroscopy proves to be a valuable technique for layer identification and monitoring the effect of strain on the optical bandgap. We demonstrate that uniaxial strains of up to 3% can be applied to 3L MoS_2_ with enhanced strain transfer efficiency, comparable to those achieved when encapsulated with polymers, and over significantly larger sample areas, thanks to the robust interaction between MoS_2_ and Au. This enables, for instance, the use of a scanning probe microscope to image topographical changes under high strain. Additionally, this methodology can be employed for many other 2D materials. Furthermore, other exfoliated materials could be transferred onto the 2D material–Au system to enhance the substrate interaction through van der Waals forces. These findings contribute to the development of strain-engineered 2D-based flexible devices with enhanced performance and functionality.

## Methods

*Sample Fabrication*. MoS_2_ was exfoliated from natural molybdenite (Molly Hill Mine, Quebec, Canada) on 250 μm thick polycarbonate substrates (Modulor GmbH) previously covered with 3 nm Ti and 6 nm Au using a home-built electron beam evaporator. With the gold-assisted exfoliation technique, large-size MoS_2_ monolayers can easily be obtained.^21^

*Optical Characterization*. Differential reflectance measurements were performed using a home-built microreflectance setup.^43^ Briefly, spectra were collected from a spot of ∼1.4 μm diameter with a Thorlabs CCS200/M fiber-coupled spectrometer (Thorlabs, Inc., Newton, New Jersey, U.S.A.) using a Motic BA310 MET-T microscope equipped with a 50× objective and an AMScope MU1803 CMOS camera.

Raman measurements were carried out with a MonoVista CRS+ system (Spectroscopy and Imaging GmbH) with 532 nm laser excitation using a 50× objective with a laser power of 0.3 mW and an integration time of 60 s. Diffraction gratings of 2400 lines/mm were used.

*X-ray Diffraction*. Texture analysis of Au-covered polycarbonate films was carried out in D8 Discover, Bruker.

*Straining Setup*. A home-built automated three-point bending apparatus was used to apply uniaxial strain to the samples. The calibration of the strain was carried out using patterned micropillars, allowing for the direct measurement of the applied strain. More details of the setup can be found in the study by Çakıroğlu et al.^27^

## Supporting Information Available

Additional optical images of the samples, differential reflectance experiments under uniaxial strain, and data-fitting details (PDF)
