# Supplementary material for: Improved Strain Transfer Efficiency in Large-Area Two-Dimensional MoS2 Obtained by Gold-Assisted Exfoliation
Source: J Phys Chem Lett. 2024 Jun 10;15(24):6355–62. doi: 10.1021/acs.jpclett.4c00855 (PMC11194808; doi:10.1021/acs.jpclett.4c00855)
Supplement: Supplementary file 1 — jz4c00855_si_001.pdf [file jz4c00855_si_001.pdf]

Supporting Information

Improved Strain Transfer Efficiency in Large-Area 2D MoS_2 Obtained by Gold-Assisted Exfoliation

Álvaro Rodríguez[1*], Onur Çakıroğlu[1], Hao Li[1], Felix Carrascoso[1], Federico Mompean[1], Mar Garcia-Hernandez[1], Carmen Munuera[1*], Andres Castellanos-Gomez[1*]

[1]Materials Science Factory, Instituto de Ciencia de Materiales de Madrid (ICMM-CSIC), C. Sor Juana Inés de la Cruz, 3, Madrid, 28049, Spain.

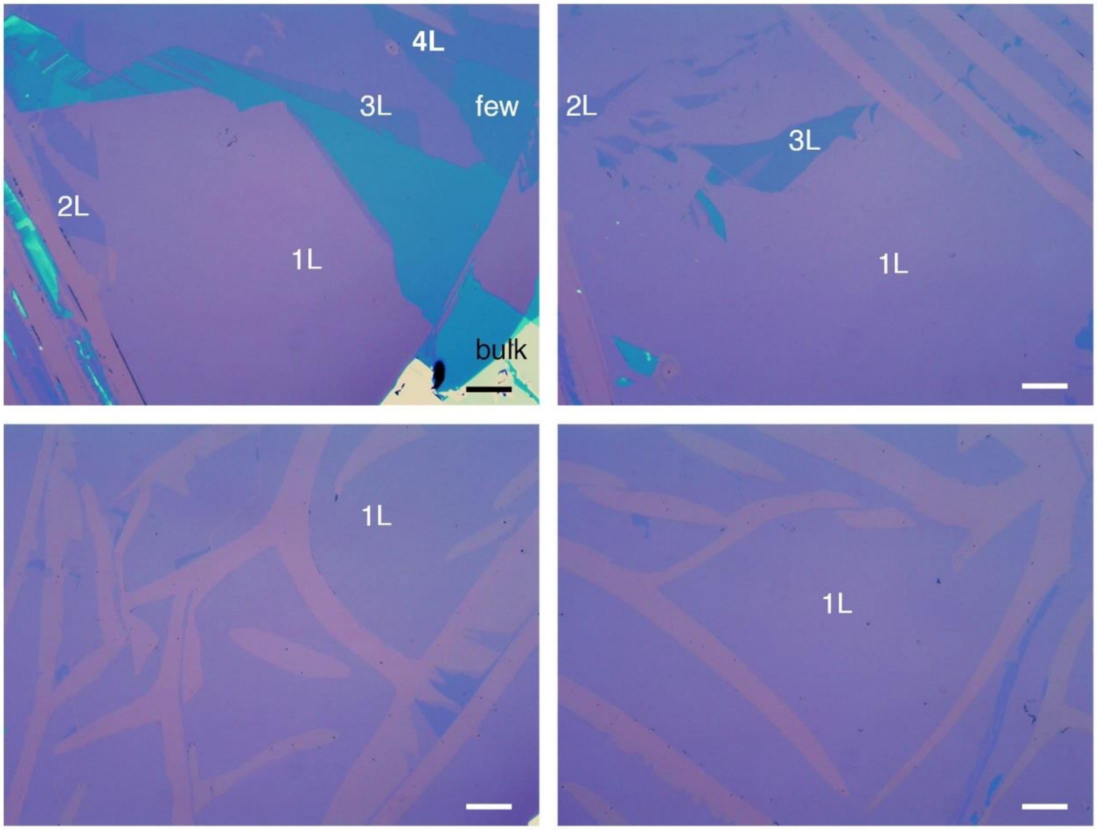

Figure S1. Additional optical images of the samples obtained by gold-exfoliation method on polycarbonate substrates.

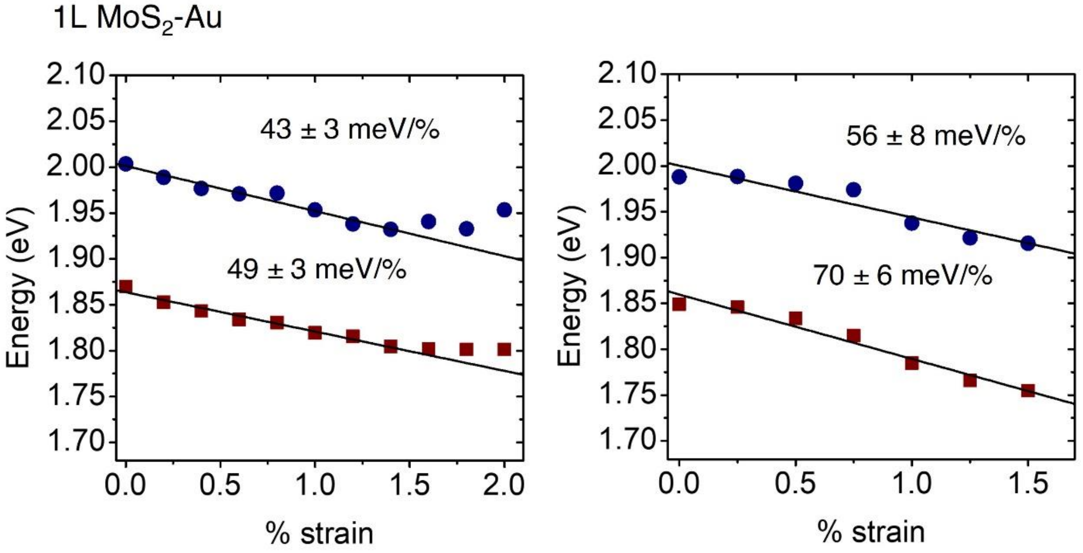

Figure S2. Gauge factors of additional 1L MoS_2-Au samples obtained from differential reflectance spectra for A (dark red squares) and B exciton (dark blue circles) energies as a function of the applied uniaxial strain.

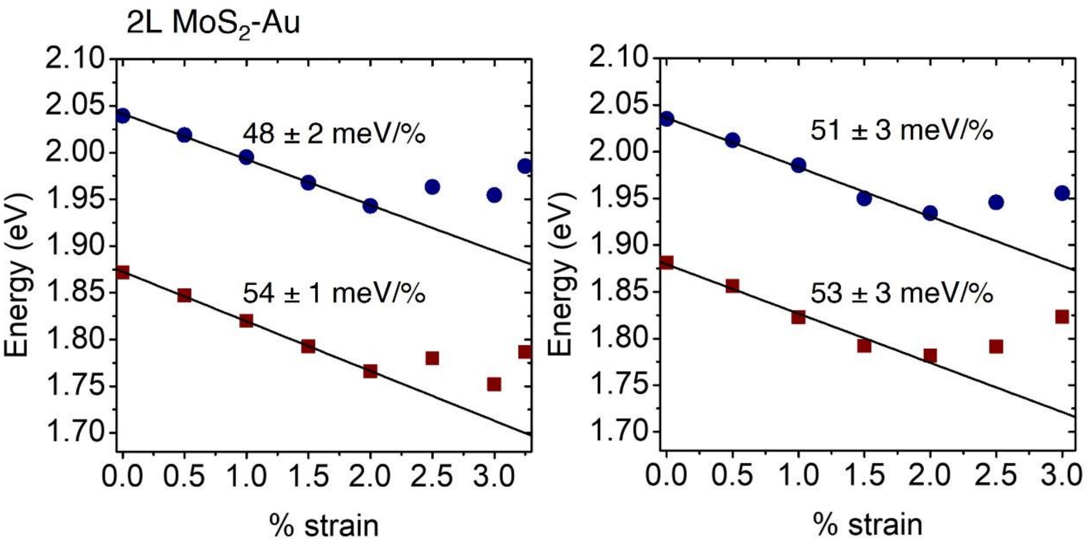

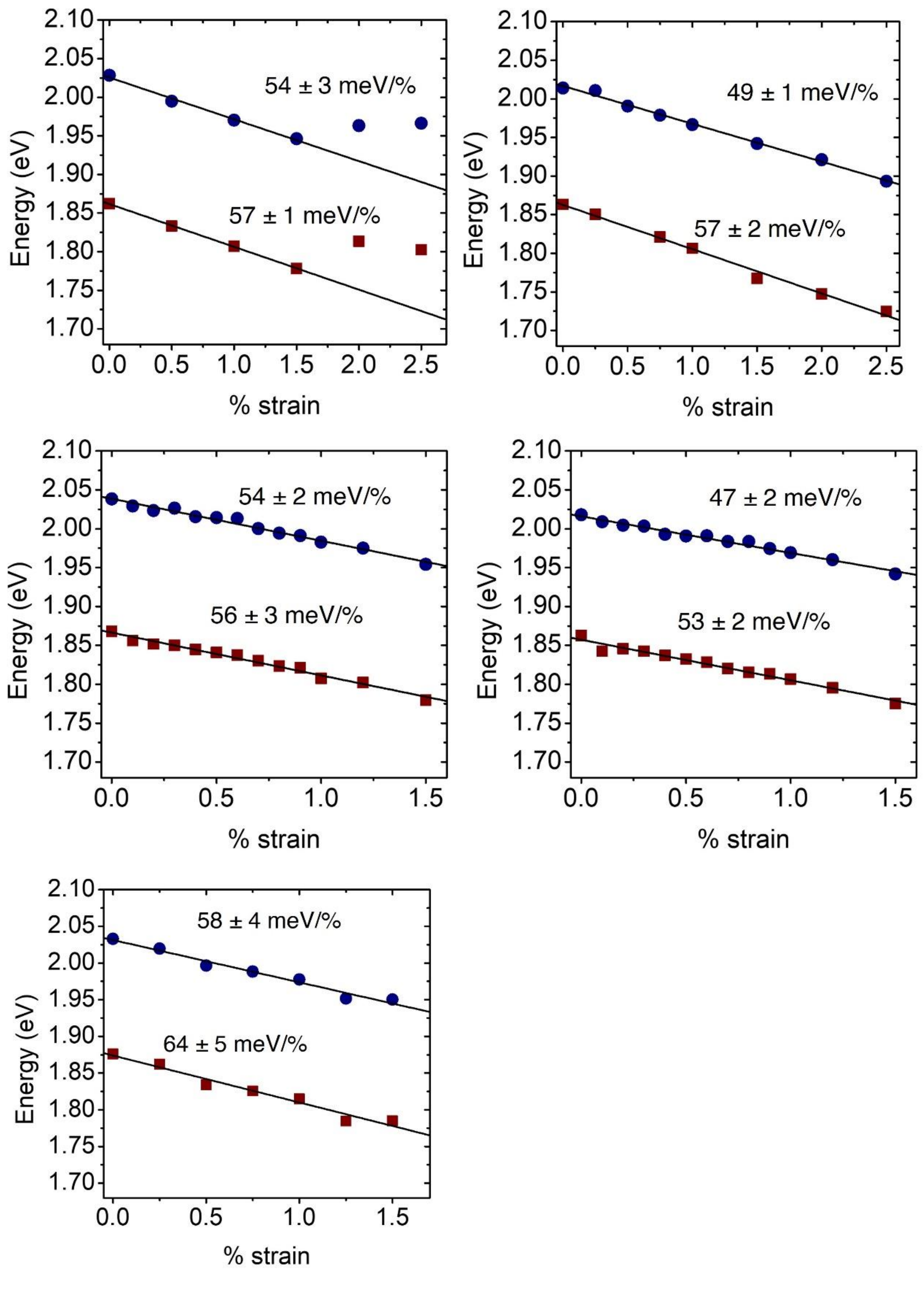

Figure S3. Gauge factors of additional 2L MoS_2-Au samples obtained from differential reflectance spectra for A (dark red squares) and B exciton (dark blue circles) energies as a function of the applied uniaxial strain.

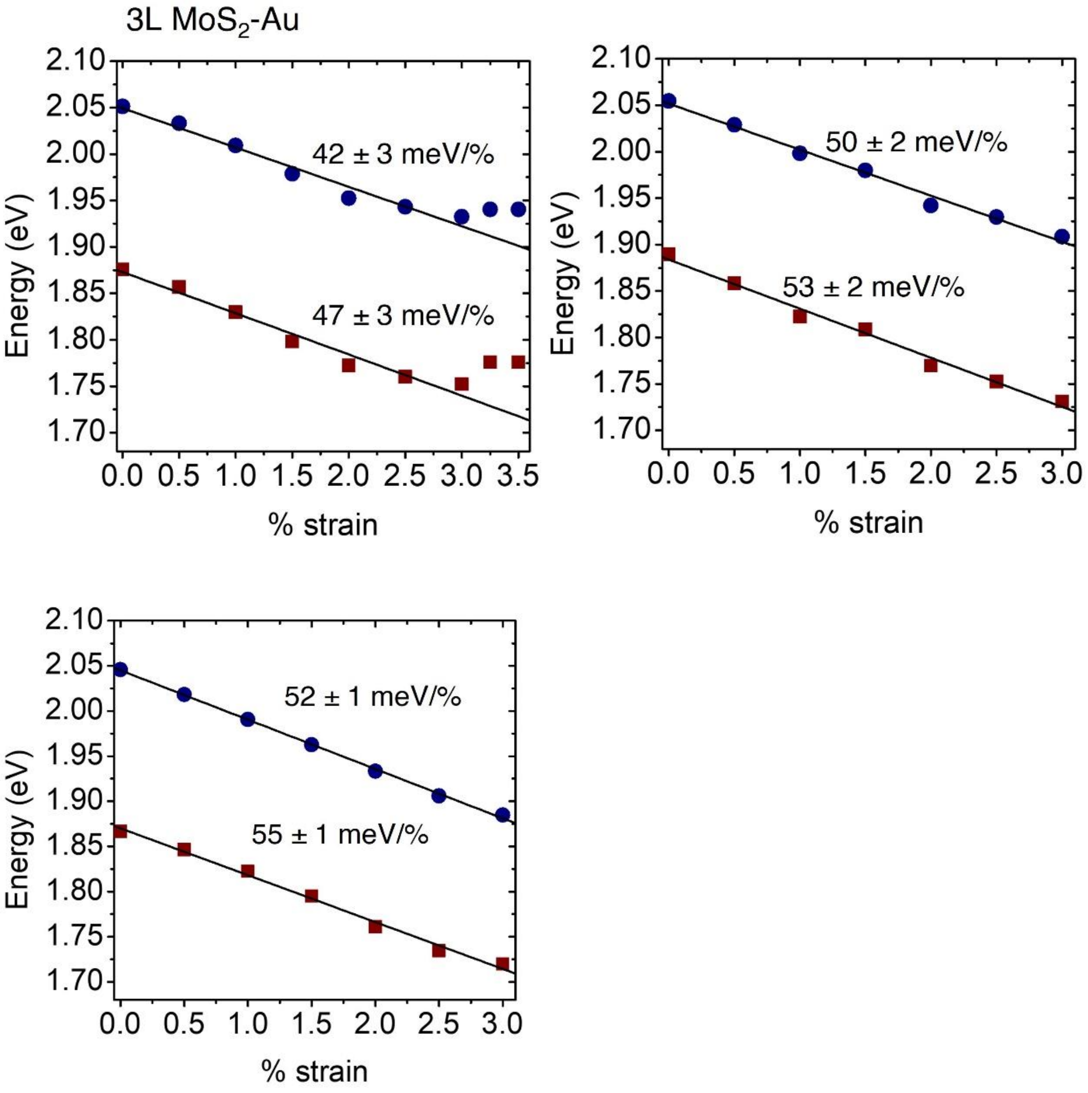

Figure S4. Gauge factors of additional 3L MoS_2-Au samples obtained from differential reflectance spectra for A (dark red squares) and B exciton (dark blue circles) energies as a function of the applied uniaxial strain.

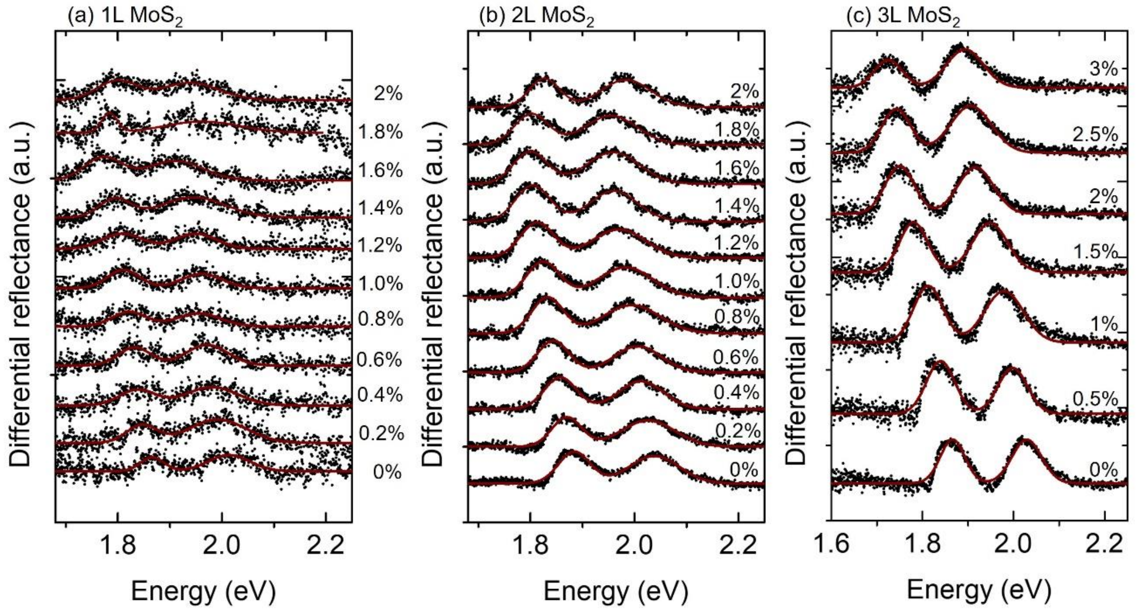

Figure S5. Fitting curves of the differential reflectance spectra for mono- (1L), bi- (2L), and tri- (3L) layer MoS2 with increasing strain. The spectra correspond to those presented in Figure 3(a-c).

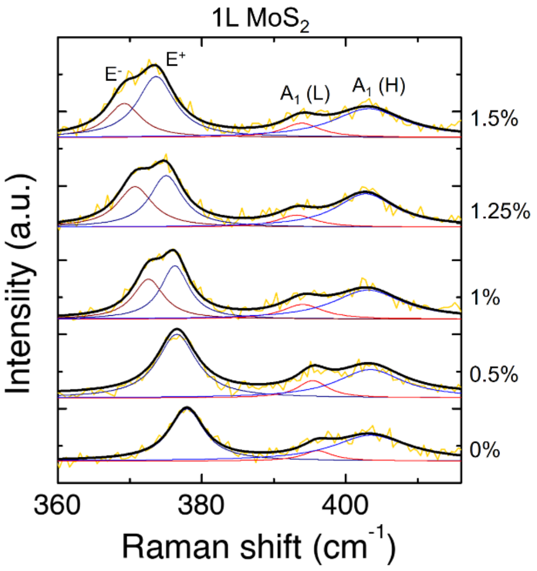

Figure S6. Deconvolution of the Raman spectra for monolayer MoS_2 with increasing strain, as indicated.

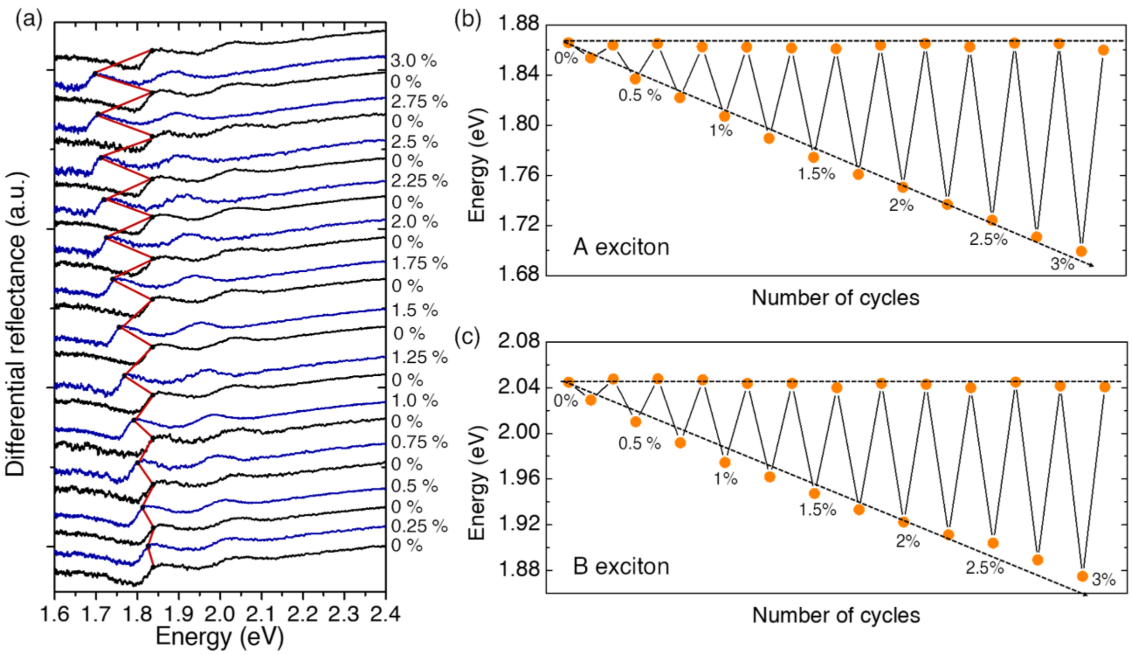

Figure S7. (a) Differential reflectance spectra of multiple strain-relaxation cycles in 3L MoS2, with strain levels indicated in the figure. (b) A exciton and (c) B exciton energy positions during the strain-relaxation cycles.
